# Radial Nerve Neuropraxis due to Compression by C-Arm Fluoroscopy in Spine Surgery: A Case Report

**DOI:** 10.1155/2020/3086787

**Published:** 2020-09-16

**Authors:** João Bragança, Matthieu Hanauer, Guillaume Racloz

**Affiliations:** Department of Orthopaedics and Traumatology, Réseau Hospitalier Neuchâtelois, Rue de la Maladière 45, 2000 Neuchâtel, Switzerland

## Abstract

**Introduction:**

Peripheral nerve injury is a well-known surgical complication related to the position of the patient. Moreover, in spine surgery, prone position for prolonged period places the patient at increased risk. The aim of this study was to report a case of a radial nerve neuropraxis due to compression by C-arm fluoroscopy during spine surgery. *Case Presentation*. An 81-year-old-female underwent a posterior spinal fixation L2-S1 due to lumbar spinal stenosis. In the recovery room, she presented an hematoma at the posterolateral part of her arm associated with a wrist drop due to radial nerve neuropraxis. The patient was referred to an occupational therapist and fully recovered four months later. After analysis of the patient positioning during the intervention, we came to the conclusion that this radial nerve injury was very possibly due to a compression by the C-arm fluoroscopy during the surgery.

**Conclusion:**

Our case describes a rare case of compression of the radial nerve during lumbar spine surgery, which is an unexpected complication as the site of the nerve injury is not at all related to the surgery itself, but to the position of the patient. Although C-arm fluoroscopy is essential, spine surgeons should be aware of this possible complication related to its use in order to avoid it.

## 1. Introduction

Peripheral nerve injury is a well-known surgical complication related to the position of the patient on the operative table. Among all types of surgery, the incidence of perioperative peripheral nerve injury varies between 0.03 and 0.1% [1], secondary to nerve stretching, compression, and sometimes both. Dorsal position with abduction and external rotation of the arm can lead to injure the brachial plexus or more commonly the ulnar nerve (28%) [1], whereas injuries of the radial nerve are rarer.

Prone position during spine surgery may last for hours, which is not a physiologic condition. This position may increase the risk of peripheral nerve injury. Although potentially harmful, these injuries can be easily avoided so long as some precautions are taken.

In literature, only few articles describe specific nerve injury in spine surgery related to patient positioning. Moreover, no case has been related to the use of a C-arm fluoroscopy so far. We present a case of a radial nerve neuropraxis due to compression by C-arm fluoroscopy on elbow in spine surgery.

## 2. Case Presentation

An 81-year-old-female presented to the emergency room after a mechanical fall of her height at home. Clinical history points out a severe neurogenic claudication in aggravation for 6 months. All complementary exams were normal at the emergency department, and, later, a magnetic resonance imaging showed lumbar spinal stenosis. After obtaining her informed consent, she sustained a posterior spinal fixation L2-S1. The surgery underwent successfully. However, in the recovery room, the patient presented a wrist drop at her right side. The clinical exam showed a hematoma at the posterolateral part of her right arm associated with a full inability to extend her right wrist and metacarpophalangeal joints without any sensitive deficit, compatible with a radial nerve injury. During her stay in our department, an intensive physiotherapy treatment was initiated. The patient left the hospital at day 7 with a 2/5 strength grade extension deficit. Rehabilitation was continued ambulatory twice a week. At 6 weeks, the patient presented a 4/6 strength grade extension deficit. Four months postoperatively, the patient had fully recovered and returned to her preinjury level.

## 3. Discussion

Perioperative peripheral nerve lesion remains a rare complication, with a reported incidence of 0.03-0.1%, as well as radial nerve injury which accounts for only 3% of all peripheral nerve injuries, all types of surgery combined [1]. In spine surgery specifically, incidence of neurological injuries related directly to the surgery rises to 5.7% [2].

The radial nerve originates from the posterior cord of the brachial plexus and courses in a spiral groove at the posterior aspect of the humeral shaft, where it may be vulnerable to injury. Then, it pierces the lateral intermuscular septum reaching the anterior compartment of the arm. According to Artico et al., the mean distances between the lateral point of crossing the posterior aspect of the humerus and the lateral and medial epicondyles are 121 (+/-13) and 125 (+/-13) mm, respectively [3].

The most common mechanism of radial nerve injury related with positioning of patients is direct compression as it gets through its humeral groove [1]. Mild nerve compression injuries often cause neuropraxis. According to Seddon et al., as no Wallerian degeneration occurs in this situation, motor deficit has generally a good prognosis to recover within 12 months [4] [5] [6].

**Figure 1:**
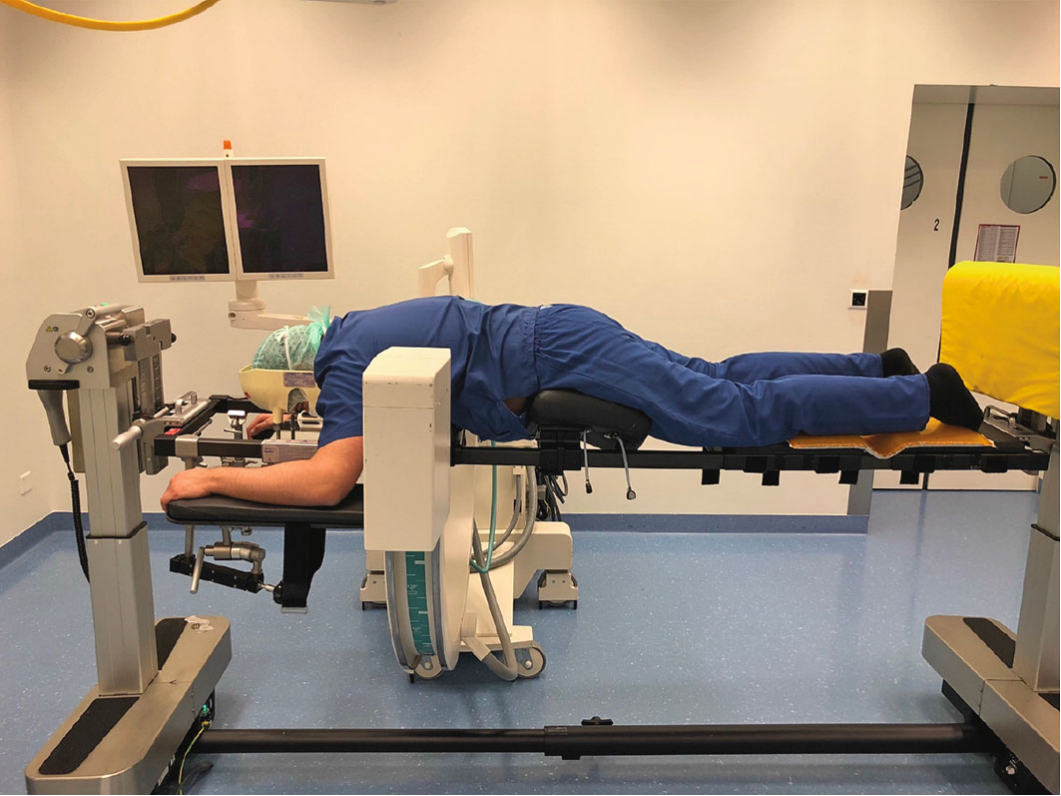
Radial nerve sustained a compression by the C-arm fluoroscopy—global view.

**Figure 2:**
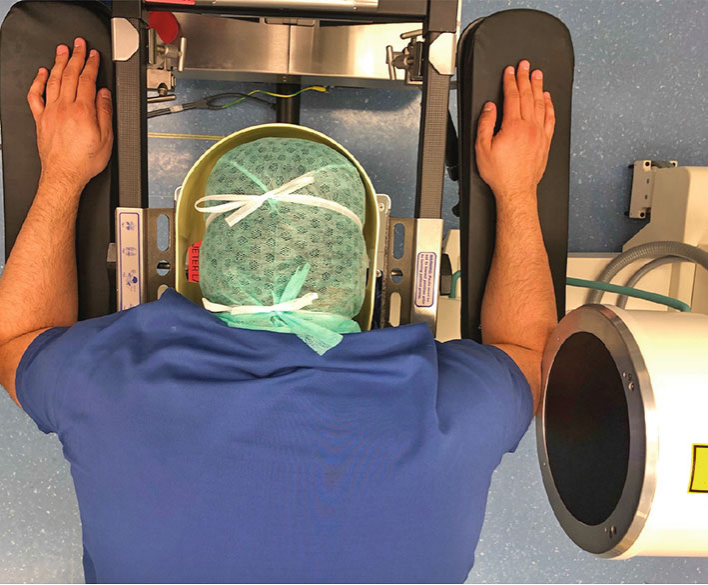
Radial nerve sustained a compression by the C-arm fluoroscopy—global view.

**Figure 3:**
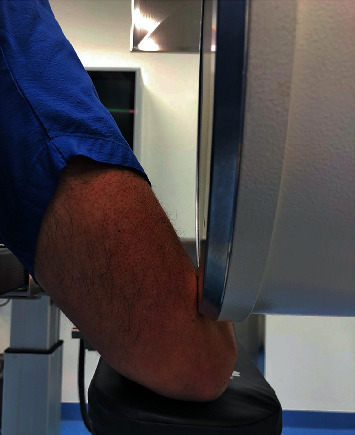
Radial nerve sustained a compression by the C-arm fluoroscopy—close view.

In our surgery, after screw implantation, the C-arm was moved towards the head to clear the operative field. At this moment, we started the implantation of four interbody cages for three hours. During this time, the C-arm compressed the radial nerve in its groove at the posterolateral aspect of the arm, as shown in Figures 1, 2, and 3.

To the best of our knowledge, we demonstrated an unexpected situation that has not been described so far, as the site of the nerve injury was not at all related to the surgery itself, but to the use of a specific instrumentation combined with the position of the patient.

During spine surgery, C-arm fluoroscopy is essential to control the good position of implanted material and requires to be moved frequently. During each utilization, the C-arm fluoroscopy is displaced to the operating field and moved towards the head of the patient permitting the surgeon to proceed to the surgery. After each manipulation, it is essential to check the C-arm fluoroscopy position, in order to avoid any compression of the arm of the patient.

According to our experience and in order to avoid such a complication, we strongly advise to verify the position of the C-arm fluoroscopy after each manipulation.

## 4. Conclusion

C-arm fluoroscopy is an essential instrumentation in spine surgery. However, the surgeon should be aware of the possible complication resulting of its utilization. Simple check of the proper position after each utilization of the C-arm fluoroscopy permits to avoid such debilitating complication and reduce patient morbidity.
